# Bacterial Complexity of Traditional Mountain Butter Is Affected by the Malga-Farm of Production

**DOI:** 10.3390/microorganisms10010017

**Published:** 2021-12-23

**Authors:** Silvia Schiavon, Mauro Paolini, Raffaele Guzzon, Andrea Mancini, Roberto Larcher, Tomas Roman Villegas, Elena Franciosi

**Affiliations:** 1 Technology Transfer Centre, Fondazione Edmund Mach (FEM), 38010 San Michele all’Adige, Italy; silvia.schiavon@fmach.it (S.S.); mauro.paolini@fmach.it (M.P.); raffaele.guzzon@fmach.it (R.G.); roberto.larcher@fmach.it (R.L.); tomas.roman@fmach.it (T.R.V.); 2 Research and Innovation Centre, Fondazione Edmund Mach (FEM), 38010 San Michele all’Adige, Italy; andrea.mancini@fmach.it

**Keywords:** raw milk, Malga, butter, bacterial counts, Illumina MiSeq, acetoin, *Lactococcus*

## Abstract

Bacteria can play different roles affecting flavors and food characteristics. Few studies have described the bacterial microbiota of butter. In the present paper, next-generation sequencing was used to determine bacterial diversity, together with aromatic characteristics, in raw cow milk butter processed by traditional fermentation, in fourteen small farms called “Malga”, located in the Trentino province (Alpine region, North-East of Italy). The physicochemical and aromatic characterization of traditional mountain butter (TMB) showed a low moisture level depending on the Malga producing the butter. Counts of lactic acid bacteria, *Staphylococci*, and coliforms, as well as diacetyl/acetoin concentrations exhibited changes according to the geographical origin of Malga and the residual humidity of butter. MiSeq Illumina data analysis revealed that the relative abundance of *Lactococcus* was higher in TMB samples with the highest values of acetoin (acetoin higher than 10 mg/kg). The traditional mountain butter bacterial community was characterized by a “core dominance” of psychrotrophic genera, mainly *Acinetobacter* and *Pseudomonas*, but according to ANCOM analysis, a complex bacterial population emerged and specific bacterial genera were able to characterize the TMB bacteria community, with their high abundance, based on the Malga producing the butter.

## 1. Introduction

A variety of traditional artisanal milk products are produced in the Trentino Alpine region (Northeastern Italy) and several studies have documented their high microbial biodiversity [1] and, more generally, the quality of traditional dairy products [2]. Most of them are produced following artisanal technologies in small-scale on-farm plants called “Malga”, which are located between 1400 and 2000 m a.s.l. Each Malga is subjected to a short opening time from the end of May until the beginning of October, when cattle from the valley are delivered to Alpine pastures for free grazing. In each Malga the milk collection and the dairy process are carried out in the same place. There are two main artisanal dairy products from cow milk in the Malga: traditional mountain cheese [1] and traditional mountain butter (TMB). Raw milk creaming and churning are the most important steps for the TMB making, when the oil-in-water emulsion is broken leading to aqueous phase separation and the formation of water-in-oil emulsion [3]. There are two main characteristics distinguishing TMB from commercial butter: no salt is added and no kind of ripening is carried out of neither the milk cream nor the butter, and the TMB is sold no later than one day after production [4]. The manufacturers have adopted these two solutions in order to limit, as much as possible, the oxidative processes that can lead to a decrease in the nutritional value and customer acceptability. Fat oxidative reactions are chemical reactions that spontaneously occur and cannot be stopped even if the storage temperature is lowered [5]. Salt is sometimes added to butter, but has been shown to be a lipid pro-oxidant factor in different foods, due to both chloride [6] and metal ion impurities that enhance its pro-oxidant effect [7]. For these reasons, the TMB is produced without salt and immediately sold and consumed.

One of the key features driving the consumer in choosing a butter is associated to a “natural image” [8], and the consumer perception is that dairy products from cows that are fed by fresh grass are “more natural” [9], and even better if the cows are kept outdoors in Alpine pastures. This perception led to an increase in the interest and consumption of TMB regarded as a “natural product”, due to its traditional production in the Malga-farms (Mfarms) located in Alpine pastures. The TMB flavor and the choice of the Malga are other important parameters that significantly affect the consumers’ satisfaction [8] and from where to acquire the butter. For these reasons, TMB became a very popular local product, manufactured “on demand” in the Malga for tourists, and catering for consumers that booked in advance the amount of butter to buy in their favorite Malga.

Compared to industrially produced butter, there are very few data regarding butter produced by milk from pasture-based feeding [10,11] and are mainly focused on chemical characterization. Thus, the aim of this work is to evaluate the quality of raw cow milk TMB, processed in fourteen representative Malga of the Trentino Alpine region from a microbiological and aromatic point of view. The microbial quality of TMB was evaluated considering the bacterial biodiversity by plate counts and next-generation sequencing technologies (NGST) [12]. Over the past decade, NGST evolved rapidly and led to an improved representation of sample biodiversity [13]. To our knowledge, published studies of food microbial ecology have rarely been performed on butter and this can be one of the first studies about it, together with the very recent works by El-Hajjaji et al. [14] and Syromyatnikov et al. [15]. The aromatic quality of TMB was evaluated by considering the diacetyl and acetoin amounts, since these compounds are the most common potent odorants in dairy products [16] that provides a buttery, sweet, and creamy odor. In particular, acetoin has an important effect on the aroma of butter due to its low perception threshold [16], and can be generated either from the reduction of diacetyl by the enzyme diacetyl reductase or from the conversion of lactose, pyruvate, or citrate by means of lactic acid bacteria (LAB) [17]. Diacetyl, is recognized as relevant to butter aroma and also as an antimicrobial agent against some pathogens associated to raw milk dairy products, such as *E. coli*, *L. monocytogenes*, and *S. aureus* [18]; therefore, the identification of factors enhancing its concentration in TMB can contribute to the improvement of the safety of this traditional food.

## 2. Materials and Methods

### 2.1. Butter Production and Sampling

In the summer season of 2020, 42 artisanal cow butter samples were collected from 14 high-mountain Mfarms, hereafter named from A to P (three biological replicates sampled in July, August, and September, respectively, from each Mfarm), located in the Trentino Alpine region (Northeastern Italy). The Mfarms are grouped into two areas of the region, the Val di Sole (West of the Trentino province) and Valsugana (East of the Trentino province). Each Mfarm produced its own TMB using the fresh, raw cows’ milk directly after spontaneous skimming was performed according to the traditional method. Only the Mfarms A, H, and O pre-maturated the milk before skimming: the raw milk was left at 8–10 °C in a plastic tank for about 18 h before skimming and the TMB production process was similar in each Mfarm. Details about the Mfarm, milk volumes, and TMB production process are reported in Table S1. Briefly, for the spontaneous skimming, the milk was gravity separated at about 12 °C in a flat steel uncovered vat. After the overnight rest of about 10 h, the skim milk fraction was drained from the bottom of the flat vat, and the top fraction left in the vat was constituted by the cream and drained after the skimmed milk but in a plastic bow. The creams were churned until the phases’ separation. Churning takes place at 12 °C in an electric churn and is achieved after vigorously shaking the product back and forth until the coalescence of the fat globules. The end of churning was identified by the sound of the butter lumps when being shaken after about 40 min. The grains of butter were washed three times with cold water (8–10 °C) in order to remove the buttermilk, kneaded by hand on a table to remove excess buttermilk, and, finally, packed into blocks of 500 g. the butter was stored in refrigerator (4 °C) for max 24 h before selling. No starter cultures or salt were added.

Three cow butter samples (approximately 0.2 kg from each 500 g block) were randomly collected from the Mfarm to have a representative picture of the production for the day. Each of the three samples was individually analyzed for microbial plate counts and chemical analysis. The three samples were pooled for the DNA extraction before the Illumina MiSeq analysis.

### 2.2. Microbiological and Physicochemical Analyses

Ten grams of each sample were shaken in 90 g of sterile peptone water (1 g/L) for 1 min at 260 rpm by a Stomacher Lab Blender 400 BA 7021 (Seward Medical, Worthing, UK); heated at 45 °C for 3 min; shaken again by a Stomacher for 1 min; decimally diluted in sterile peptone water; and plated onto the following agar media: de Man, Rogosa and Sharpe (MRS) agar for cultivating mesophilic *lactobacilli*, incubated in anaerobic conditions (in a jar with an “Anaerogen” anaerobic system) for 48 h at 30 °C; M17 agar, for cultivating mesophilic *lactococci*, incubated in aerobic conditions for 48 h at 30 °C; plate count agar (PCA) with skimmed milk (10 g/L) for the total bacterial count (TBC), in aerobic conditions for 24 h at 30 °C; violet red bile agar (VRBA) for counting enterobacteria, following the overlay method as suggested by the manufacturer’s instructions, for 24 h at 37 °C; kanamycin aesculin azide agar (KAA) for the count of *enterococci* aerobically incubated for two days at 37 °C, Baird Parker agar (BPA) for the count of coagulase-positive *staphylococci*, including 5% egg yolk and tellurite; and, lastly, aerobic incubation at 37 °C for 48 h. An enzymatic–fluorogenic method was used for the detection of *Escherichia coli* by means of 4-methylumbelliferyl-β-D-glucuronide (MUG), which, when hydrolyzed by β-D-glucuronidase, releases 4-methylumbelliferone (4-MU), a fluorogenic molecule that emits fluorescence when irradiated with an ultraviolet light wavelength [19]. Approximately 97% of *E. coli* are positive for this enzyme, while the same enzyme is not as prevalent in others from the *Enterobacteriaceae* family [20]. MUG was already present as a supplement in the VRBA medium. After the incubation of the VRBA plates at 37 °C for 24 h, the fluorescence readings were recorded with an excitation wavelength of 305 nm and the emission wavelengths in the visible range, as suggested by the manufacturer’s instructions. All culture media and anaerobic systems were purchased from Oxoid (Thermo Fisher Scientific, Waltham, MA, USA). The moisture was evaluated using IDF recommended standards ISO 3727-1:2001 [21].

### 2.3. DNA Extraction, Miseq Library Preparation, and Illumina Sequencing

For the total genomic DNA extraction, between 0.4 and 0.6 g of butter were used. Genomic DNA was extracted following the CTAB method, according to the instructions [22] of Pirondini et al., and quantified by a Nanodrop8800 Fluorospectrometer (Thermo Fisher Scientific).

Amplicon library preparation, the quality and quantification of pooled libraries, and pair-end sequencing using the Illumina MiSeq system were carried out, at the sequencing platform in Fondazione Edmund Mach (FEM, San Michele all’Adige, Italy). Briefly, the total genomic DNA was amplified using primers specific to the bacterial V3–V4 region [23,24] of the 16S rRNA gene (*Escherichia coli* positions 341 to 805). Each sample was amplified by PCR using 25 μL reaction with 1 μM of each primer. The PCR reactions were executed by the GeneAmp PCR System 9700 (Thermo Fisher Scientific). The amplification products were checked on 1.5% agarose gel and purified using the Agencourt AMPure XP system (Beckman Coulter, Brea, CA, USA), following the manufacturer’s instructions. Subsequently, a second PCR was used to apply dual indices and the Illumina sequencing adapters Nextera XT Index Primer (Illumina, San Diego, CA, USA). The amplicon libraries were purified using the Agencourt AMPure XP system (Beckman), and the quality control was performed on a Typestation 2200 platform (Agilent Technologies, Santa Clara, CA, USA). Finally, all barcoded libraries were pooled in an equimolar ratio and sequenced on an Illumina^®^ MiSeq (PE300) platform (MiSeq Control Software 2.5.0.5 and Real-Time Analysis software 1.18.54.0).

### 2.4. Illumina Data Analysis and Sequences Identification by QIIME2

Raw paired-end FASTQ files were demultiplexed using idemp (https://github.com/yhwu/idemp/blob/master/idemp.cpp, accessed on 17 December 2021) and imported into Quantitative Insights Into Microbial Ecology, Qiime2, version 2020.11 [25]. The sequences were quality-filtered, trimmed, de-noised, and merged using DADA2 [26]. The chimeric sequences were identified and removed via the consensus method in DADA2. The representative sequences were aligned with MAFFT and used for phylogenetic reconstruction in FastTree using plugins alignment and phylogeny [27]. Taxonomic and compositional analyses were carried by using the plugins feature classifier (https://github.com/qiime2/q2-feature-classifier, accessed on 17 December 2021). A pre-trai, accessed ned Naive Bayes classifier based on the Greengenes gg_13_5_otus.tar.tgz Operational Taxonomic Units (OTUs) database (http://greengenes.secondgenome.com/?prefix=downloads/greengenes_database/gg_13_5/, accessed on 17 December 2021), which had been previously trimmed to the V4 region of 16S rDNA, bound by the 341F/805R primer pair, was applied to paired-end sequence reads to the generate taxonomy tables.

### 2.5. GC–MS Analysis of Acetoin and Diacetyl

The procedure used for the acetoin and diacetyl extraction from butter was based on the method reported by Gokce et al. [28]. In a 15 mL tube, 4 g of butter was melted at 40 °C for 5 min. Then, 0.2 g of MgSO4 and 10 µL of internal standard solution (2, 3-pentadione in methanol at 20 g/L) were added. The tube was shaken vigorously for 5 min. Acetoin, diacetyl, and 2,3-pentadione were extracted adding 4 mL of acetone, shaken for 5 min, and finally centrifuging at 4000 rpm for 5 min. The upper phase was filtered through a 0.20 µm filter and transferred in vial for the GC–MS analysis.

For the quantification, a butter sample was spiked with a standard solution of diacetyl and acetoin (10 g/L in methanol) at three concentration levels (3, 6, and 12 µg/g) and extracted as reported. The diacetyl and acetoin analysis was performed using an Agilent Intuvo 9000 fast GC system coupled with an Agilent 7000 Series Triple Quadrupole MS equipped with an electron ionization source (EI) (70 eV, 50 μA). Separation was obtained by injecting 2 μL in split mode (1:10) into a CP-Wax 57 CB capillary column (50 m, 0.32 mm id × 0.20 μm film thickness) with He as the carrier gas at a flow of 0.8 mL/min. The oven temperature was programed starting at 40 °C for 2 min, raised to 150 °C by 7 °C/min, and finally raised to 200 °C by 20 °C/min, and held at this temperature for 2 min. The injector and transfer line temperature were both set at 250 °C, whereas the source temperature was set at 230 °C. The mass spectrometer acquisition was performed in selected-ion monitoring mode (SIM) by recording the abundance of the ions having mass-to-charge ratio of 44–86–87 *m*/*z* for diacetyl, 43–45–88 *m*/*z* for acetoin, and 29–57–100 *m*/*z* for 2,3-pentadione. The dwell time was set to 50 ms and the solvent delay was set for 4 min. the data acquisition and analysis were performed using the MassHunter Workstation software supplied by the manufacturer. The quantification was based on the peak area using one target ion and two qualifier ions.

### 2.6. Statistical Analysis

The data regarding the microbiological counts were analyzed as means expressed in log CFU/g. Statistical analysis was performed, analyzing the technology, the month, and the Mfarm of production as independent variables, and the bacterial plate counts, acetoin, and diacetyl concentrations as dependent variables. For the bacterial counts and diacetyl content, statistical analysis (one-way Anova with post-hoc Tukey HSD test) was performed on the entire set of samples (*n* = 42 for 5 biological replicates) without the application of data transformation, as the data met the assumption of normality (Shapiro–Wilk W test) and homoscedasticity (Levene’s test).

The Pearson’s correlation test was used to determine the relations between the aromatic variables (moisture, acetoin, and diacetyl) and the different bacterial populations in butter. All the tests on the plate counts, acetoin, and diacetyl amounts were performed using the STATISTICA data analysis software system, version 9.1 (TIBCO Software Inc. (2017). Statistica (data analysis software system), version 13. Tulsa, OK, USA, http://statistica.io, accessed on 17 December 2021).

Differences in diversity indices (OTUs number and Shannon’s diversity index) of the different samples were tested by the Kruskal–Wallis test using a plug-in implemented in QIIME2. The statistical significance of the communities among all samples was assessed via the non-parametric PERMANOVA (permutational multivariate analysis of variance) by means of a plug-in implemented in QIIME2. For the differential abundance test, the taxonomy information for each OTU sequence was provided using the ANCOM method by means of a plug-in implemented in QIIME2.

The geographical discrimination was assessed by splitting the dataset into two groups, Val di Sole and Valsugana, and applying the principal component analysis (PCA) algorithm using the statistical significant variables (Tukey’s HSD test). The PCA was performed using the R version 4.0.5 software package.

The correlation between the diacetyl/acetoin concentration and the microbiological/physicochemical parameters was obtained by applying a partial least squares regression (PLSR), while the predictive models for the diacetyl and acetoin concentrations were obtained with a nonlinear regression analysis.

The PLSR analysis and the predictive models were both performed using XLSTAT software.

## 3. Results and Discussion

### 3.1. Microbiological Physicochemical and Aromatic Characteristics of TMB

The present work proposes, for the first time, a survey of the main variables of TMB production in Italy, summarized in Table S1. We considered, as the main sources of variability, the geographical location (Valsugana and Val di Sole), the numbers of cows managed by each Mfarm (ranging from 20 to 70 cows), the amount of milk available for TMB production (ranging from 150–700), the churn nominal volume (ranging from 20 to 500 L), and the manufacturing material used for the TMB knead table (wood, Teflon, or stainless steel). All these variables can affect the TMB microbiota, and the aromatic and physicochemical properties. The microbiological quality of the TMB samples is presented in Table 1.

The TBC indicates the load of the total viable aerobic mesophilic bacteria that was always very high, ranging from 5.8 to 8.2 Log CFU/g (butter samples collected from E and C Mfarms, respectively). TMB samples also showed high LAB counts: the *lactobacilli* count ranged from 4.4 to 7.0 Log CFU/g (samples collected from F and O Mfarms, respectively); the *lactococci* counts, with the exception of Mfarm E (mean value was 4.5 Log CFU/g), were very similar in the TMB samples, ranging from 6.2 to 7.1 Log CFU/g and always higher than the *lactobacilli* counts. Similar results were reported in an earlier study on traditional butters [29] and are considered very high, when compared with a previous work by Samet-Bali et al. [30] on traditional Tunisian butters. The high TBC and LAB counts can be attributed to the absence of milk pasteurization and salt. The natural milk bacteria load can increase due to the effect of both the spontaneous separation and churning process on the breaking up of the bacterial clumps, which increase their number [31].

In butter production, the *Staphylococci* and enterobacteria counts are generally used as important hygiene and contamination indicators. Despite the application of preventive measures (refrigeration and hygienic practice) during its production and distribution, the butter can be prone to contamination by *Staphylococci* and coliforms [32]. The coliforms count was very variable, in a range from 3.5–3.9 Log CFU/g (samples collected from E and D Mfarms, respectively) to 6.9 Log CFU/g (samples collected from both H and O Mfarms), and all the samples tested had levels of *E. coli* contamination below 2 Log CFU/g (data not shown). Coagulase-positive *Staphylococci* in food products can produce enterotoxins, and cause staphylococcal food poisoning outbreaks. The count of *Staphylococci*, like the coliforms, was very variable and in a range from 2.7–2.9 Log CFU/g (samples collected from D and M Mfarms, respectively) to 5.8 Log CFU/g (samples collected again from both H and O Mfarms).

The moisture values of the TMB made in the 14 different Mfarms are shown in Table 1. Five Mfarms (B, D, E, I, and P) showed moisture contents consistent with the previously reported data on unsalted fresh butter made from cow milk [33]. The other nine Mfarms showed significantly lower moisture contents, probably due to the longer kneading step after washing the butter granules, allowing a more efficient removal of the buttermilk, and, consequently, a lower moisture level in the fresh TMB. By correlating the moisture with bacterial plate counts, no significance (*p* always higher than 0.05) and no linear trend (R^2^ always lower than 0.4) were evident, probably because the salt absence made the water content a non-limiting factor for the microbial growth.

Acetoin and diacetyl are generally described as essential components of butter aroma compounds. In TMB, the diacetyl concentration mean value was always lower than 1 mg/kg (Table 2) and, in half of the TMB samples, the diacetyl concentration was lower than 0.1 mg/kg.

This was in accordance with a previous study, in which this compound was not found in traditional fresh butter stored for less than 20 days [28], probably due to the need for time for the bacterial conversion of milk citric acid by strains of *Lactococcus lactis* subsp. *lactis* [34]. The average concentration of acetoin was 13.9 ± 24.4 mg/kg (Table 2), following the same trend of diacetyl, for which the diacetyl concentration was lower than 0.1 mg/kg, and the acetoin was recorded at very low values that were always lower than 1 mg/kg. In the other TMB samples, the acetoin concentration was in a range of 5.14 mg/kg (at Mfarm L) to 7.36 mg/kg (at Mfarm O), according to a previous study on traditional fresh butter [26]. In 3 Mfarms the diacetyl/acetoin mean values were significantly higher (63.88, 32.84, and 32.60 at Mfarms H, N, and P, respectively) than in other TMB samples, and one order of magnitude higher than in previous works on butter [28,35]. Comparing both the diacetyl and acetoin with bacterial plate counts, no significance (*p* always higher than 0.05) and no linear trend (R^2^ close to 0) were evident.

### 3.2. Characteristics of Sample Sequence and Alpha Diversity

With the exception of seven butter samples from seven different Mfarms, the extracted DNA was successfully amplified in the bacterial V3-V4 16S rRNA gene region. From the 35 TMB samples, 2,012,558 sequences (average length of 413 bp) were obtained.

Alpha diversity analysis revealed that the values of the OTUs number and Shannon’s index varied a lot among the different samples, but never significantly (Table 3).

The higher the OTUs number and Shannon’s index, the more bacterial diversity and richness are present in the sample. All the samples presented numbers of OTUs in the range of 266 (TMB at Mfarm B) and 490 (TMB at Mfarm C), and the values of Shannon’s index in the range of 6.94 (TMB at Mfarm B) and 8.21 (TMB at Mfarm L), which are very high when compared with the values obtained from the other butter samples analyzed by means of NGST [14,36]. This great difference in bacterial biodiversity cannot be due to different steps in the butter production, in agreement with El-Hajjaji et al. [14]. In this work, butter was produced by spontaneous skimming of raw unsalted milk kept at temperatures lower than 14 °C, and the cream was also characterized by lower bacterial biodiversity. Therefore, we speculated that the sources of this richness could be the milk used for the production and their geographical source, as already shown by Carafa et al. [37] in one Mfarm, located in the Trentino area, with very high bacterial biodiversity in the raw milk.

### 3.3. Taxonomic Composition of the Bacterial Community

**Figure 1:**
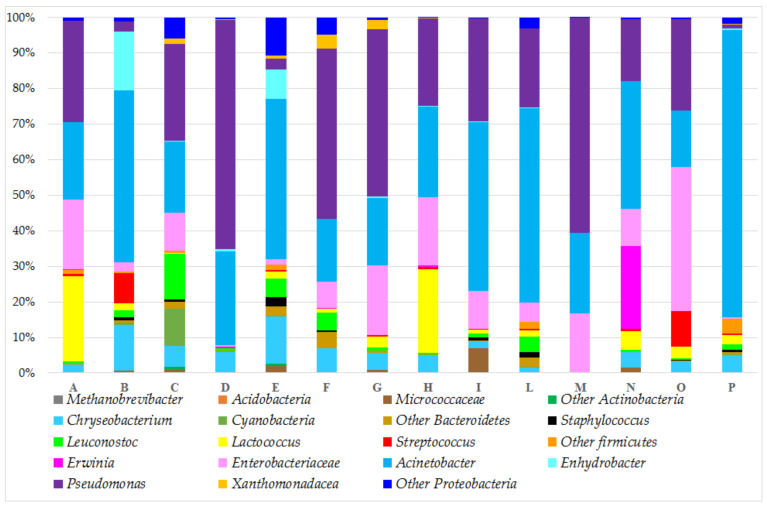
Relative abundances (%) of the main taxa groups (genus level or above) of bacterial sequences from different TMB samples using Illumina MiSeq. Each bar is the mean value of 3 samples collected in different Mfarms located in the Trentino province (for the interpretation of the references to color in this figure legend, the reader is referred to the web version of this article).

The use of 16S rRNA gene sequencing identified a total of 14 phyla and 112 genera across all samples. The relative abundance of bacterial community proportions at the phylum, genus, and species levels are shown in Figure 1 (only phylotypes with a relative abundance of at least >0.5% are shown).

The main phyla were *Methanobrevibacter*, *Acidobacteria*, *Actinobacteria*, *Bacteroidetes*, *Firmicutes*, and *Proteobacteria*, that were present in all the samples, in accordance with prior investigations on the bacterial biodiversity of Alpine milk collected from a single Malga located in Trentino [37]. *Proteobacteria* was the dominant phylum in all the samples, in agreement with the findings reported for other traditional butters [36] or butter obtained by cream ripened at 4 °C [14] that, analyzed by NGST, were most populated by *Proteobacteria* and contained low amounts of *Firmicutes*. *Firmicutes* is the dominant phylum in traditional dairy products [2]; conversely, in TMB, the *Firmicutes* was always lower than 13%. We observed only two Mfarms (A and H) producing TMB with a higher relative abundance of *Firmicutes* (24.9% and 23.8%, respectively). Reads belonging to *Cyanobacteria* phylum likely come from the amplification and sequencing of plant chloroplasts that originated from *Cyanobacteria* [38]. The main taxa detected in TMB that alone described more than 85% of the total bacterial population were as follows (Figure 1): *Chriseobacterium* (an average relative abundance of 4.7%), *Lactococcus* (5.5%), *Enterobacteriaceae* (13.7%), *Acinetobacter* (35.4%), and *Pseudomonas* (27.9%). These bacterial taxa are consistent with previous data on raw cow milk cream samples from spontaneous creaming collected in the same area [39]. *Pseudomonas* and *Acinetobacter*, as psychrotrophic microorganisms, grow well even at 4 °C [40,41] and are commonly present as dominant Gram-negative bacteria in dairy milk [37,39,42]. Conversely to *Pseudomonas* and *Acinetobacter*, the relative abundance of *Lactococcus* in TMB samples was much lower, probably because refrigeration had an effect on the *Lactococcus* growth [42]. Unlike the majority of the samples, *Lactococcus* showed a high relative abundance in TMB from Mfarms A and H (relative abundances were always higher than 20%); we speculated that this could be an effect of the milk pre-maturation before skimming that allowed an overgrowth of *Streptococcaceae* bacteria. In fact, the Mfarms in which the milk pre-maturation was performed before skimming (A, H, and O, Table S1) showed a total abundance of *Streptococcaceae* (*Lactococcus* and *Streptococcus* species) higher than the other Mfarms. The total *Lactococcus* and *Streptococcus* relative abundances were 22.89%, 23.09%, and 13.11% in Mfarms A, H, and O, respectively (Table S2); conversely, in the other Mfarms, in which the TMB was produced without milk pre-maturation, the total relative abundance of the *Lactococcus* and *Streptococcus* species was never higher than 5.91% (with only the exception of Mfarm B, with a relative abundance of *Lactococcus* and *Streptococcus* species of 10.36%).

The PERMANOVA analysis revealed no significant (*p* > 0.08) bacterial composition difference among TMB samples, suggesting that neither the Malga of production nor the month of sampling had any significant effect on the TMB bacterial community.

Alongside the dominant taxa, other bacteria with an average relative abundance of < 1% were detected. The analyses for the family and genus level using ANCOM models revealed that most of these minor bacterial taxa were the drivers of the differences in the bacterial diversity of TMB per Mfarm (Table S2). *Micrococcaceae* (driver at Malga I) and *Enhydrobacter* (driver at Malga B and E) were never previously observed in butter. Both of these two taxa were already found present in Malga milk [37], which can explain their occurrence in butter. *Erwinia* is another uncommon genus that was detected in this study. Its relative abundance was always <1% with the exception of Mfarm N, in which it was the dominating taxa and driver characterizing the TMB samples (relative abundance of 23.1%). *Erwinia* are herb-associated bacteria found in high relative abundances in some Alpine herbs in the Trentino highland in the summer season [43] and can, thus, be introduced into raw milk by a cross-contamination of pasture herbs microbiota. *Leuconostoc* (driver at Mfarm C) and *Streptococcus* (driver at both Mfarms B and O) are the most common LAB genera found in milk and dairy products besides *Lactococcus* [2]. The complex bacterial community in butter samples with single bacterial taxa characterizing different productions was already found in a previous work on traditional butter [44]. We speculated that the microbiota of the butter is composed of a dominant “core population” common to all butters depending more on the process of production. In our case study, this “core population” is constituted by the psychrotrophic genera *Pseudomonas* and *Acinetobacter* due to the low temperatures of the process never reaching higher than 12 °C. Besides the “core population”, there are minorities of bacteria present in smaller amounts, only in some butters, which are the drivers of geographical characterization and probably derive from environmental contamination. The homogeneity of the dominant population did not allow significant differences emerging from a beta diversity analysis; conversely, the ANCOM analysis was able to highlight the differences among the TMB productions due to the minority bacterial populations.

Looking at bacteria taxa involved in a pathogenic role for human health, we did not find any OTUs belonging to the most dangerous representatives of foodborne bacteria, such as *Salmonella* spp., *Listeria* spp., and *E. coli*. *Staphylococcus* OTUs relative abundances were always lower than 1% with the exception of Mfarms E (2.4%) and L (1.6%). These results are apparently not in accordance with the *Staphylococci* microbial counts because Mfarm E showed the lowest counts of *Staphylococci*. Considering the *Staphylococci* count in relation to TBC, the value was high and in accordance with MiSeq Illumina data relative abundances. Another similar discrepancy between plate counts and Illumina data was recorded for the *Enterococcus* species, whose counts were high by plate counts but never higher than 0.4% by MiSeq Illumina data. These results are in agreement with a previous work by Carafa et al. [45], in which the *Enterococci* data by Illumina were not fitting with the plate counts in cheese samples, and confirmed the importance of always performing a quantitative analysis of the viable bacterial cells because the only Illumina data analysis can lead to mistakes in overestimation or underestimation.

### 3.4. Geographical Discrimination: Val di Sole vs. Valsugana

**Figure 2:**
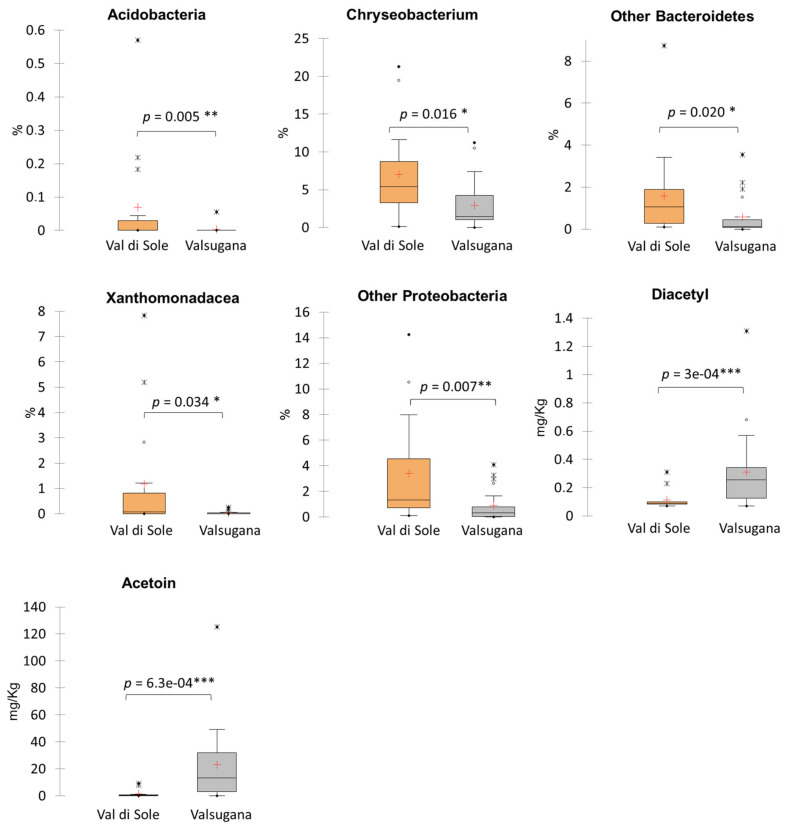
Box plot of the relative abundances (%) of *Acidobacteria, Chryseobacterium, other Bacteroidetes, Xanthomonadaceae,* other *Proteobacteria,* and the concentration of diacetyl/acetoin measured in the TMB of the two valleys in Trentino (Val di Sole, *n* = 17; Valsugana *n* = 25). Significance level: * *p* ≤ 0.05 significant; ** *p* ≤ 0.01 very significant; *** *p* ≤ 0.001 highly significant.

The whole dataset was divided into the two main geographical locations (Val di Sole and Valsugana), and a statistical analysis using the microbiological and physicochemical parameters and Illumina data was performed. The seven variables that showed significant differences between the two areas of origin (*p* < 0.05 Anova with post-hoc Tukey HSD) were reported in Figure 2.

The TMB sampled from Valsugana Mfarms showed a significantly higher concentration of diacetyl and acetoin than the samples from Val di Sole, and a higher variability inside the pool of data. The samples recognized as outliers (Figure 2) were produced in A and H Mfarms, respectively. We speculated that the milk pre-maturation step, performed by both Mfarms A and H before TMB production, can be a source of high variability affecting the acetoin/diacetil production.

**Figure 3:**
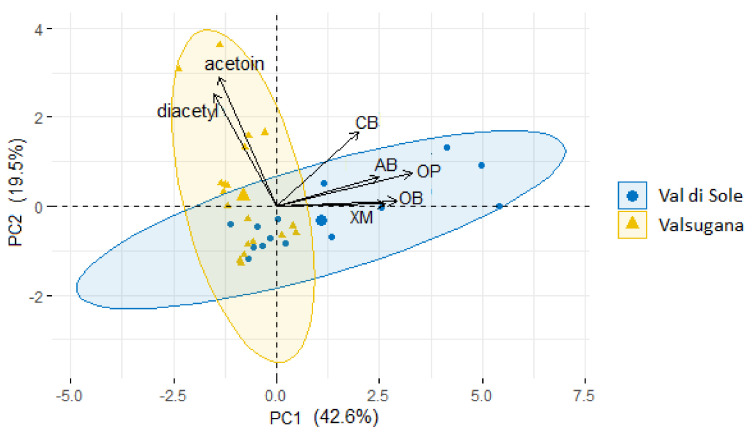
Plots of loadings and scores for principal components 1 and 2 (PC1 and PC2) obtained by principal component analysis (PCA).

Considering the Illumina data analysis, the box plots in Figure 2 indicate a correlation between the Mfarm location and the relative abundances of *Acidobacteria*, *Chryseobacterium*, other *Bacteroidetes*, *Xanthomonadaceae*, other *Proteobacteria*, and the concentration of diacetyl/acetoin quantified in the TMB. The TMB sampled in Val di Sole Mfarms showed significantly higher relative abundances of these five bacterial phylotypes than the samples from Valsugana (Figure 2). Additionally, principal component analysis (PCA) using the seven discriminant variables of *Acidobacteria* AB; *Chryseobacterium* CB; other *Bacteroidetes* OB; *Xanthomonadaceae* XM; other *Proteobacteria* OP; and the concentration of diacetyl/acetoin was also performed. As shown in Figure 3, the 2 principal components (PC1 and PC2) described 62.1% of the total variance (TV); the first component PC1, explains the 42.6% while the second component, PC2, explains the 19.5% of the TV.

The main variables contributing to the PC1 dimension were the relative abundances of *Acidobacteria*, *Chryseobacterium*, other *Bacteroidetes*, *Xanthomonadaceae*, and other *Proteobacteria*; conversely, the main variables contributing to the PC2 dimension were the concentration of diacetyl and acetoin. The PCA analysis did not allow for the clustering of the TMB samples according the two geographical locations, Val di Sole and Valsugana, in agreement with the beta diversity analysis of the Illumina data, confirming that the geographical location did not affect the microbial differences among the Mfarms.

### 3.5. Predictive Model of Diacetyl and Acetoin Concentration

**Figure 4:**
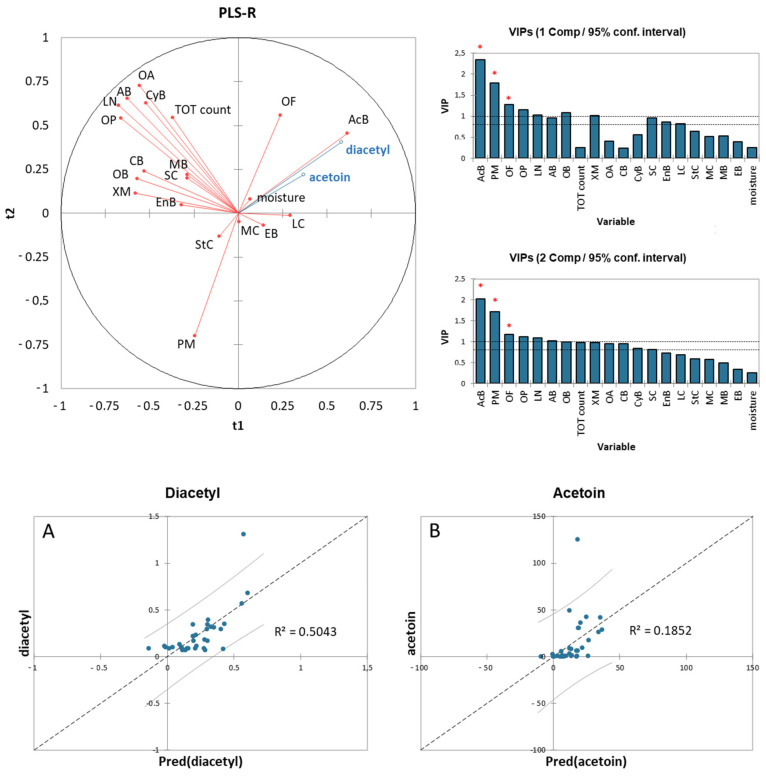
Partial least squares regression (PLSR) analysis results: VIP (variable importance in prediction) scores and correlation plots between actual and predicted values of (**A**) diacetyl and (**B**) acetoin. Note: MB: *Methanobrevibacter*; AB: *Acidobacteria*; MC: *Micrococcaceae*; OA: other *Actinobacteria*; CB: *Chryseobacterium*; CyB: *Cyanobacteria*; OB: other *Bacteroidetes*; SC: *Staphylococcus*; LN: *Leuconostoc*; LC: *Lactococcus*; StC: *Streptococcus*; OF: other *Firmicutes*; EB: *Enterobacteriaceae*; AcB: *Acinetobacter*; EnB: *Enhydrobacter*; PM: *Pseudomonas*; XM: *Xanthomonadaceae*; and OP: other *Proteobacteria*. Significance level: * *p* ≤ 0.05 significant.

The correlation between the diacetyl/acetoin concentration and the Illumina data, TBC, and moisture of the TMB samples was performed using a partial least squares regression (PLSR) cross-validation procedure, in order to build a predictive model. The results of the PLSR analysis are presented in Figure 4.

The entire dataset was used as a calibration model, but the variables with the highest importance in prediction scores (VIP: red asterisks in Figure 4) were the relative abundances of *Acinetobacter*, *Pseudomonas*, and other *Firmicutes*. The PLS predictive models for the content of diacetyl and acetoin in the TMB samples, considering the VIPs as variables for the quantitative analysis of variation, provided the determination coefficients of 0.5043 and 0.1842 for diacetyl and acetoin, respectively.

**Figure 5:**
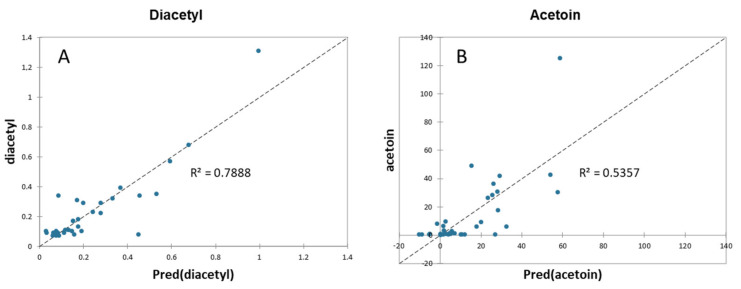
Correlation plots between the actual and predicted values of (**A**) diacetyl and (**B**) acetoin with a polynomial function of the second order.

In order to improve the correlation between the measured and predicted concentrations of diacetyl/acetoin, a polynomial function model was used. The new predictive models were built using the variables showing higher positive indices with diacetyl and acetoin in the correlation matrix (Table S3). The accounted variables were the relative abundances of *Lactococcus*, other *Firmicutes*, *Enterobacteriaceae*, *Acinetobacter*, TBC, and moisture. The predictive models for the diacetyl and acetoin concentrations have been built by fitting the data with a polynomial function of the second order (Figure 5).

These new models were characterized by a coefficient of determination (R^2^) higher than the one obtained with the PLS models for both diacetyl and acetoin. By fitting the data with a polynomial function of the third order, the correlation between the measured and predicted concentrations improved, reaching a coefficient of determination (R^2^) of 0.9280 and 0.6571 for diacetyl and acetoin, respectively.

## 4. Conclusions

We characterized and compared the bacterial composition of different traditional fermented butter products collected from 14 Malga-farms in the Alpine area of the Trentino region. We showed that butter is a good substrate for the growth of bacteria from a broad range of taxa in very high amounts, even if all the steps of production are carried out at low temperatures. We were able to identify the bacterial species by both high-throughput sequencing and traditional plating on a nutritive medium. Our results showed great biodiversity among the TMB production of the Malga considering the physicochemical parameters, acetoin amount, plate counts, and bacterial community observed by means of MiSeq Illumina. Traditional mountain butter samples contained complex bacterial communities: the predominant bacterial taxa all belonged to *Proteobacteria* phylum and were *Acinetobacter* and *Pseudomonas*, but, according to the ANCOM analysis, we also found that each Malga production was characterized by individual bacterial taxa able to drive the difference in the TMB production of each Malga. Additionally, the procedure of the pre-maturation of the milk could induce an increase in the abundance of *Lactococcus* and *Streptococcus* in the butter and considering that, by means of ANCOM analysis, *Lactococcus* presence was related to the high acetoin amount in the butter, the milk pre-maturation practice could be considered to increase the quantity of this important aromatic compound in butter.

## Figures and Tables

**Table 1:** Microbiological characteristics and moisture of traditional mountain butter (TMB) samples produced in the Malga, in the Trentino Alpine region. Abbreviation: TBC, total bacteria counts. Plate counts (Log colony forming units (CFU)/g) and moisture data (g/100 g) are expressed as the mean ± SD (*n* = 3 biological replicates). For each column, the bacterial count, and moisture values with a, b, and c superscripts are significantly different (*p* < 0.05, one-way Anova with post-hoc Tukey HSD).

Mfarm	TBC	*Lactococci*	*Lactobacilli*	*Staphylococci*	Coliforms	*Enterococci*	Moisture
A	7.1 ± 0.59 ^b^	7.1 ± 0.73 ^c^	6.9 ± 1.58 ^bc^	4.2 ± 0.80 ^b^	6.3 ± 1.69 ^bc^	4.4 ± 0.15 ^b^	14.3 ± 0.18 ^b^
B	6.1 ± 0.43 ^a^	6.2 ± 0.66 ^b^	5.2 ± 0.25 ^a^	4.7 ± 0.61 ^bc^	4.3 ± 1.59 ^a^	4.4 ± 0.06 ^b^	16.8 ± 1.30 ^bc^
C	8.2 ± 0.85 ^c^	7.1 ± 1.08 ^bc^	5.9 ± 1.26 ^ab^	3.7 ± 1.83 ^ab^	5.3 ± 1.94 ^b^	3.9 ± 1.36 ^ab^	12.9 ± 0.41 ^ab^
D	6.7 ± 0.01 ^a^	6.4 ± 0.67 ^b^	5.6 ± 0.01 ^b^	2.7 ± 0.00 ^a^	3.9 ± 0.13 ^a^	3.5 ± 0.21 ^a^	17.5 ± 5.66 ^bc^
E	5.8 ± 0.25 ^a^	4.5 ± 0.28 ^a^	5.2 ± 0.66 ^a^	3.5 ± 0.25 ^a^	3.5 ± 1.67 ^a^	3.4 ± 0.32 ^a^	14.3 ± 0.60 ^bc^
F	6.8 ± 1.80 ^ab^	6.4 ± 1.86 ^bc^	4.4 ± 1.20 ^a^	3.8 ± 1.88 ^ab^	5.1 ± 1.18 ^b^	3.1 ± 1.38 ^ab^	13.0 ± 0.44 ^ab^
G	7.0 ± 0.80 ^b^	7.1 ± 0.95 ^c^	5.7 ± 0.11 ^b^	3.9 ± 0.18 ^b^	4.5 ± 0.63 ^a^	4.4 ± 0.67 ^b^	13.4 ± 0.86 ^ab^
H	7.9 ± 0.16 ^c^	6.8 ± 1.48 ^bc^	6.9 ± 0.46 ^c^	5.0 ± 1.64 ^bc^	6.9 ± 0.66 ^c^	5.0 ± 0.78 ^c^	9.3 ± 1.61 ^a^
I	7.0 ± 0.52 ^b^	6.2 ± 1.95 ^bc^	5.8 ± 0.20 ^b^	4.6 ± 1.77 ^bc^	6.5 ± 1.39 ^bc^	5.3 ± 0.46 ^c^	18.3 ± 3.34 ^c^
L	6.6 ± 0.99 ^ab^	6.6 ± 1.06 ^bc^	4.5 ± 0.97 ^a^	4.2 ± 0.85 ^b^	4.1 ± 1.37 ^a^	2.6 ± 0.60 ^a^	13.9 ± 0.93 ^b^
M	7.3 ± 0.39 ^b^	6.3 ± 1.05 ^bc^	5.9 ± 0.52 ^b^	2.9 ± 1.79 ^ab^	6.4 ± 1.57 ^bc^	4.7 ± 1.97 ^bc^	12.0 ± 0.91 ^ab^
N	7.6 ± 0.28 ^b^	6.9 ± 1.28 ^bc^	6.8 ± 0.48 ^c^	3.6 ± 0.58 ^ab^	6.8 ± 1.00 ^bc^	4.6 ± 0.38 ^b^	11.6 ± 0.84 ^ab^
O	7.5 ± 0.23 ^b^	7.1 ± 0.12 ^c^	7.0 ± 0.17 ^c^	5.8 ± 1.08 ^c^	6.9 ± 0.03 ^c^	5.5 ± 0.01 ^c^	12.4 ± 0.06 ^ab^
P	6.5 ± 0.78 ^ab^	6.6 ± 1.08 ^bc^	4.9 ± 0.14 ^a^	3.0 ± 1.73 ^ab^	4.0 ± 1.16 ^a^	2.8 ± 0.74 ^a^	18.6 ± 1.29 ^c^
TOT	6.8 ± 0.88	5.6 ± 1.62	5.5 ± 1.15	2.9 ± 1.79	4.5 ± 1.10	3.7 ± 1.54	14.1 ± 3.1

**Table 2:** Extraction recoveries of acetoin and diacetyl in butter samples collected from different farms at spiked levels by the GC–MS method. Data (mg/kg) are expressed as the mean ± SD (n = 3 biological replicates). For each column, the bacterial count values with a, b, c, and d superscripts are significantly different (*p* < 0.05, one-way Anova with post-hoc Tukey HSD).

MFarm	Diacetyl	Acetoin
A	0.203 ± 0.122 ^b^	5.95 ± 4.66 ^b^
B	0.085 ± 0.007 ^a^	0.25 ± 0.09 ^a^
C	0.090 ± 0.000 ^a^	0.35 ± 0.22 ^a^
D	0.095 ± 0.007 ^a^	0.59 ± 0.00 ^a^
E	0.085 ± 0.021 ^a^	0.20 ± 0.01 ^a^
F	0.090 ± 0.000 ^a^	0.49 ± 0.47 ^a^
G	0.090 ± 0.014 ^a^	0.59 ± 0.63 ^a^
H	0.317 ± 0.025 ^c^	63.88 ± 4.17 ^c^
I	0.190 ± 0.139 ^bc^	7.09 ± 6.87 ^b^
L	0.173 ± 0.006 ^b^	5.14 ± 1.60 ^b^
M	0.087 ± 0.015 ^a^	0.24 ± 0.12 ^a^
N	0.310 ± 0.050 ^c^	32.84 ± 9.38 ^c^
O	0.175 ± 0.064 ^b^	7.36 ± 2.27 ^b^
P	0.853 ± 0.399 ^d^	32.60 ± 8.42 ^c^
TOT	0.226 ± 0.239	13.9 ± 24.4

**Table 3:** Richness (observed Operational Taxonomic Units (OTUs) = ObsOTUs) and Shannon’s diversity index (averages ± standard deviations) of TMB samples.

MFarm	ObsOTUs	Shannon’s Index
A	403 ± 171	7.75 ± 0.808
B	266 ± 176	6.94 ± 0.310
C	490 ± 197	8.17 ± 0.563
D	395 ± 28	7.83 ± 0.053
E	356 ± 194	7.82 ± 0.915
F	369 ± 118	7.58 ± 0.514
G	449 ± 64	7.91 ± 0.021
H	464 ± 121	8.10 ± 0.640
I	396 ± 24	7.98 ± 0.189
L	475 ± 56	8.21 ± 0.178
M	420 ± 101	7.70 ± 0.842
N	459 ± 120	8.07 ± 0.595
O	445 ± 27	7.86 ± 0.390
P	419 ± 40	7.97 ± 0.302
TOT	418 ± 116	7.87 ± 0.613

## Data Availability

The data generated by Illumina sequencing were uploaded in the NCBI Sequence Read Archive (SRA) and are available under Ac. PRJNA762229.

## Supplementary Materials

The following are available online at https://www.mdpi.com/article/10.3390/microorganisms10010017/s1. Table S1, the main features of TMB productive processes in each Mfarm considered in this work. Table S2, bacterial taxa composition (in mean relative abundance) of TMB samples as revealed by Illumina high-throughput sequencing analysis. Bold numbers represent the relative abundances of the taxa driving the bacterial diversity in the TMB of each Mfarm, according to ANCOM analysis. Only taxa with an averaged relative abundance > 0.5%, are shown. MC: *Micrococcaceae*; OA: other *Actinobacteria*; CB: *Chryseobacterium*; CyB: *Cyanobacteria*; SC: *Staphylococcus*; LN: *Leuconostoc*; LC: *Lactococcus*; StC: *Streptococcus*; AcB: *Acinetobacter*; EB: *Enterobacteriaceae*; EnB: *Enhydrobacter*; PM: *Pseudomonas*; and XM: *Xanthomonadaceae*. Table S3, correlation matrix between the diacetyl/acetoin concentrations and the bacterial taxa composition, total counts and moisture. MB: *Methanobrevibacter*; AB: *Acidobacteria*; MC: *Micrococcaceae*; OA: other *Actinobacteria*; CB: *Chryseobacterium*; CyB: *Cyanobacteria*; OB: other *Bacteroidetes*; SC: *Staphylococcus*; LN: *Leuconostoc*; LC: *Lactococcus*; StC: *Streptococcus*; OF: other *Firmicutes*; EB: *Enterobacteriaceae*; AcB: *Acinetobacter*; EnB: *Enhydrobacter*; PM: *Pseudomonas*; XM: *Xanthomonadaceae*; and OP: other *Proteobacteria*.
