# Supplementary material for: Bacterial Complexity of Traditional Mountain Butter Is Affected by the Malga-Farm of Production
Source: Microorganisms. 2021 Dec 23;10(1):17. doi: 10.3390/microorganisms10010017 (PMC8778680; doi:10.3390/microorganisms10010017)
Supplement: Supplementary file 1 [file microorganisms-10-00017-s001.zip › microorganisms-1517059-supplementary.pdf]

Table S1. Main features of TMB productive process in each Mfarm considered in this work.

ID Code	Mfarm	Location	Number of cows	Volume of processed milk (L)	Churn nominal volume (L)	Manufacture Material of knead table
**A***	**Cloz**	Val di Sole	50	300	40	Stainless steel
B	Strino	Val di Sole	70	700	200	Teflon
C	Cercen	Val di Sole	40	500	60	Wood
D	Cespedè Samocleva	Val di Sole	45	500	500	Wood
E	Paludè Caldesa	Val di Sole	30	500	50	Wood
F	Senage Bolentina	Val di Sole	55	500	40	Wood
G	Valcomasine	Val di Sole	36	500	50	Teflon
**H**	**Cagnon di sotto**	Valsugana	45	550	80	Wood
I	Casabolenga	Valsugana	20	150	20	Wood
L	Setteselle	Valsugana	26	300	60	Wood
M	Valfontane	Valsugana	45	300	100	Wood
N	Valsolero	Valsugana	44	300	100	Wood
**O**	**Montagna Granda**	Valsugana	35	400	100	Wood
P	Zochi	Valsugana	50	500	30	Stainless steel

In **bold** the Mfarms carring out the milk pre–maturation before skimming. * Malga Cloz has been inserted into the Val di Sole, even if it is far about 15 kilometers, because the TMB production process is totally similar to the one adopted by the farmers in Val di Sole.

Table S2. Bacterial taxa composition (in mean relative abundance) of TMB samples as revealed by Illumina high−throughput sequencing analysis. Bold numbers represent the relative abundances of the taxa driving the bacterial diversity in TMB of each Mfarm according to ANCOM analysis. Only Taxa with an averaged relative abundance > 0.5 %, are shown. MC: *Micrococcaceae*; CB: *Chryseobacterium*; CyB: *Cyanobacteria*; SC: *Staphylococcus*; LN: *Leuconostoc*; LC: *Lactococcus*; StC: *Streptococcus;* AcB: *Acinetobacter*; EB: *Enterobacteriaceae;* EnB: *Enhydrobacter*; PM: *Pseudomonas*; XM: *Xanthomonadaceae.*

MFarm	MC	CB	CyB*	SC	LN	LC	StC	Erwinia	EB	AcB	EnB	PM	XM
**A**	0.106	2.153	0.000	0.000	0.452	**23.272**	0.616	0.198	18.989	21.001	0.128	27.572	0.018
**B**	0.561	12.692	0.437	0.756	1.941	1.889	**8.469**	0.000	2.514	47.319	**16.224**	2.685	0.043
**C**	0.529	6.285	**11.121**	0.546	**13.855**	0.092	0.056	0.000	11.215	21.253	0.369	29.000	1.449
**D**	0.059	5.897	0.063	0.000	0.442	0.156	0.000	0.448	0.263	26.373	0.593	64.425	0.034
**E**	1.735	13.168	0.168	2.392	5.211	1.864	0.557	0.041	1.379	44.378	**8.008**	3.124	0.822
**F**	0.136	6.787	0.000	0.486	5.000	1.087	0.029	0.185	7.424	17.731	0.000	48.125	3.920
**G**	0.774	4.674	0.000	0.070	0.704	2.900	0.434	0.113	18.676	18.186	0.356	45.168	2.594
**H**	0.024	4.709	0.000	0.031	0.337	**23.124**	0.569	0.401	18.733	24.911	0.215	24.232	0.048
**I**	**6.805**	2.082	0.000	0.966	1.039	1.104	0.181	0.000	10.528	46.647	0.302	28.394	0.062
**L**	0.149	1.345	0.087	1.626	4.267	1.533	0.513	0.000	5.274	53.655	0.316	21.832	0.017
**M**	0.000	0.000	0.000	0.000	0.000	0.032	0.000	0.000	16.369	22.421	0.000	59.882	0.000
**N**	1.551	4.261	0.000	0.000	0.275	5.340	0.569	**23.143**	10.438	35.519	0.000	17.372	0.010
**O**	0.000	3.216	0.000	0.306	0.438	3.114	**9.994**	0.052	40.173	15.673	0.000	25.427	0.128
**P**	0.113	4.826	0.180	0.682	1.491	2.559	0.419	0.000	0.400	81.049	0.390	1.233	0.055

* Reads belonging to *Cyanobacteria* phylum are probably coming from amplification and sequencing of plant chloroplast

Table S3. Correlation matrix between the diacetyl\acetoin concentration and the bacterial taxa composition, total count and moisture. MB: *Methanobrevibacter;* AB: *Acidobacteria;* MC: *Micrococcaceae*; OA: Other *Actinobacteria*; CB: *Chryseobacterium*; CyB: *Cyanobacteria*; OB: Other *Bacteroidetes*; SC: *Staphylococcus*; LN: *Leuconostoc*; LC: *Lactococcus*; StC: *Streptococcus;* OF: Other *Firmicutes*; EB: *Enterobacteriaceae*; AcB: *Acinetobacter*; EnB: *Enhydrobacter*; PM: *Pseudomonas*; XM: *Xanthomonadaceae;* OP: Other Proteobacteria.

**Variables**	**TBC**	**MB**	**AB**	**MC**	**OA**	**CB**	**CyB**	**OB**	**SC**	**LN**	**LC**	**StC**	**OF**	**EB**	**AcB**	**EnB**	**PM**	**XM**	**OP**	**Moisture**	**Diacetyl**	**Acetoin**
**TBC**	**1**	−0.083	0.753	−0.064	0.753	0.057	0.894	0.014	−0.056	0.710	0.119	−0.067	−0.040	0.221	−0.290	−0.125	−0.119	0.139	0.283	−0.300	−0.039	0.179
**MB**	−0.083	**1**	0.250	0.133	0.379	0.496	−0.026	0.092	−0.035	0.005	−0.098	−0.074	−0.035	−0.159	0.035	0.338	−0.183	0.073	0.664	−0.014	−0.092	−0.097
**AB**	0.753	0.250	**1**	0.023	0.920	0.348	0.895	0.269	0.365	0.883	−0.142	−0.042	0.132	−0.060	−0.145	0.161	−0.259	0.244	0.711	−0.022	−0.172	−0.168
**MC**	−0.064	0.133	0.023	**1**	0.020	−0.047	0.000	−0.101	0.140	−0.008	−0.114	−0.101	−0.127	0.133	0.141	0.027	−0.258	−0.018	0.013	0.171	−0.085	−0.100
**OA**	0.753	0.379	0.920	0.020	**1**	0.403	0.877	0.224	0.188	0.819	−0.181	−0.106	0.186	−0.048	−0.100	0.095	−0.261	0.246	0.734	−0.008	−0.082	−0.053
**CB**	0.057	0.496	0.348	−0.047	0.403	**1**	0.182	0.247	0.154	0.264	−0.263	0.324	0.005	−0.202	−0.188	0.729	−0.175	0.232	0.520	0.235	−0.128	0.095
**CyB**	0.894	−0.026	0.895	0.000	0.877	0.182	**1**	0.162	0.122	0.883	−0.104	−0.044	0.104	0.043	−0.214	0.001	−0.157	0.244	0.471	−0.040	−0.098	−0.102
**OB**	0.014	0.092	0.269	−0.101	0.224	0.247	0.162	**1**	0.466	0.575	−0.174	−0.091	0.213	−0.275	−0.053	0.075	−0.101	0.731	0.639	−0.061	−0.188	−0.200
**SC**	−0.056	−0.035	0.365	0.140	0.188	0.154	0.122	0.466	**1**	0.457	−0.208	0.084	0.400	−0.297	0.204	0.227	−0.278	0.072	0.395	0.260	−0.140	−0.220
**LN**	0.710	0.005	0.883	−0.008	0.819	0.264	0.883	0.575	0.457	**1**	−0.185	−0.051	0.236	−0.121	−0.151	0.072	−0.215	0.469	0.681	−0.003	−0.173	−0.195
**LC**	0.119	−0.098	−0.142	−0.114	−0.181	−0.263	−0.104	−0.174	−0.208	−0.185	**1**	−0.028	−0.014	0.132	−0.192	−0.106	−0.116	−0.155	−0.216	−0.348	0.136	0.160
**StC**	−0.067	−0.074	−0.042	−0.101	−0.106	0.324	−0.044	−0.091	0.084	−0.051	−0.028	**1**	−0.092	0.214	−0.165	0.609	−0.233	−0.104	−0.122	0.044	−0.109	−0.121
**OF**	−0.040	−0.035	0.132	−0.127	0.186	0.005	0.104	0.213	0.400	0.236	−0.014	−0.092	**1**	−0.289	0.422	−0.051	−0.337	−0.078	0.243	0.276	0.353	0.027
**EB**	0.221	−0.159	−0.060	0.133	−0.048	−0.202	0.043	−0.275	−0.297	−0.121	0.132	0.214	−0.289	**1**	−0.287	−0.249	−0.234	0.038	−0.270	−0.430	−0.067	0.284
**AcB**	−0.290	0.035	−0.145	0.141	−0.100	−0.188	−0.214	−0.053	0.204	−0.151	−0.192	−0.165	0.422	−0.287	**1**	−0.048	−0.574	−0.258	−0.017	0.304	0.582	0.156
**EnB**	−0.125	0.338	0.161	0.027	0.095	0.729	0.001	0.075	0.227	0.072	−0.106	0.609	−0.051	−0.249	−0.048	**1**	−0.281	−0.030	0.255	0.219	−0.154	−0.152
**PM**	−0.119	−0.183	−0.259	−0.258	−0.261	−0.175	−0.157	−0.101	−0.278	−0.215	−0.116	−0.233	−0.337	−0.234	−0.574	−0.281	**1**	−0.035	−0.279	−0.015	−0.384	−0.215
**XM**	0.139	0.073	0.244	−0.018	0.246	0.232	0.244	0.731	0.072	0.469	−0.155	−0.104	−0.078	0.038	−0.258	−0.030	−0.035	**1**	0.467	−0.118	−0.186	−0.171
**OP**	0.283	0.664	0.711	0.013	0.734	0.520	0.471	0.639	0.395	0.681	−0.216	−0.122	0.243	−0.270	−0.017	0.255	−0.279	0.467	**1**	−0.011	−0.180	−0.241
**Moisture**	−0.300	−0.014	−0.022	0.171	−0.008	0.235	−0.040	−0.061	0.260	−0.003	−0.348	0.044	0.276	−0.430	0.304	0.219	−0.015	−0.118	−0.011	**1**	0.223	−0.234
**Diacetyl**	−0.039	−0.092	−0.172	−0.085	−0.082	−0.128	−0.098	−0.188	−0.140	−0.173	0.136	−0.109	0.353	−0.067	0.582	−0.154	−0.384	−0.186	−0.180	0.223	**1**	0.483
**Acetoin**	0.179	−0.097	−0.168	−0.100	−0.053	0.095	−0.102	−0.200	−0.220	−0.195	0.160	−0.121	0.027	0.284	0.156	−0.152	−0.215	−0.171	−0.241	−0.234	0.483	**1**
